# Evolution of research in health geographics through the International Journal of Health Geographics (2002–2015)

**DOI:** 10.1186/s12942-016-0032-1

**Published:** 2016-01-20

**Authors:** Sandra Pérez, Vincent Laperrière, Marion Borderon, Cindy Padilla, Gilles Maignant, Sébastien Oliveau

**Affiliations:** UMR ESPACE 7300, University of Nice Sophia, Nice, France; UMR ESPACE 7300, University of Aix-Marseille, Aix-en-Provence, France; DSET-GS, School of Public Health, Rennes, France

**Keywords:** Health geographics, Geographic space, Spatial analysis, Lexical analysis

## Abstract

Health geographics is a fast-developing research area. Subjects broached in scientific literature are most varied, ranging from vectorial diseases to access to healthcare, with a recent revival of themes such as the implication of health in the Smart City, or a predominantly individual-centered approach. Far beyond standard meta-analyses, the present study deliberately adopts the standpoint of questioning space in its foundations, through various authors of the *International Journal of Health Geographics*, a highly influential journal in that field. The idea is to find space as the common denominator in this specialized literature, as well as its relation to spatial analysis, without for all that trying to tend towards exhaustive approaches. 660 articles have being published in the journal since launch, but 359 articles were selected based on the presence of the word “Space” in either the title, or the abstract or the text over 13 years of the journal’s existence. From that database, a lexical analysis (tag cloud) reveals the perception of space in literature, and shows how approaches are evolving, thus underlining that the scope of health geographics is far from narrowing.

## Background

Health Geographics is a relatively recent field of research. Indeed, although the link between man’s health and his environment has been underlined in medical sciences since Hippocrates, it took a long time for geography to consider that studying health facts was interesting and justified. A proper current of health geographics only emerged in the years 1970–1980. Research is flourishing and concerns themes as varied as the spread of vectorial diseases, access to healthcare, a space’s potential to be or not favorable to health, or looking for environmental determinants in the occurrence of a pathology. Spatial analysis, through the study of closeness, spread, spatial interaction, self-correlation, interpolation, progress and accessibility, is naturally at the center of this research. We wanted to find out how research work carried out on this theme has evolved with the passing time. To do so, we have opted for analyzing the way space is broached in the articles of the *International Journal of Health Geographics*, a benchmark journal on health geographics and geo-informatics fields. The material for this study was the corpus of articles published between the date when the journal was created and the present day.

## Data collection methods

The idea was to select articles including the word “space” either in the title, or the abstract or the text. 359 articles fit that request in the period spreading from the time the journal was created by Boulos [4] to the present day (September 2015). Subsequently, the title and abstract were integrated into a utility software enabling one to carry out a lexical analysis of the corpus, essentially from the most frequent words (TagCrowd) [1–3]. We only integrated the articles’ titles and abstracts into the utility, because we considered that they were good indicators of the main words used in the text, bearing in mind that they synthesize the article’s object. Representation is by means of a tag cloud. Words with the same root as *space*—like *spatial*—are merged in our request. We requested that the hundred most used words appear, as well as their frequency. We broke up the analysis period (13 years) into three temporally homogeneous sub-periods (2002–2005, 2006–2010 and 2011–2015).

## Results

### (2002–2005)

**Fig. 1:**
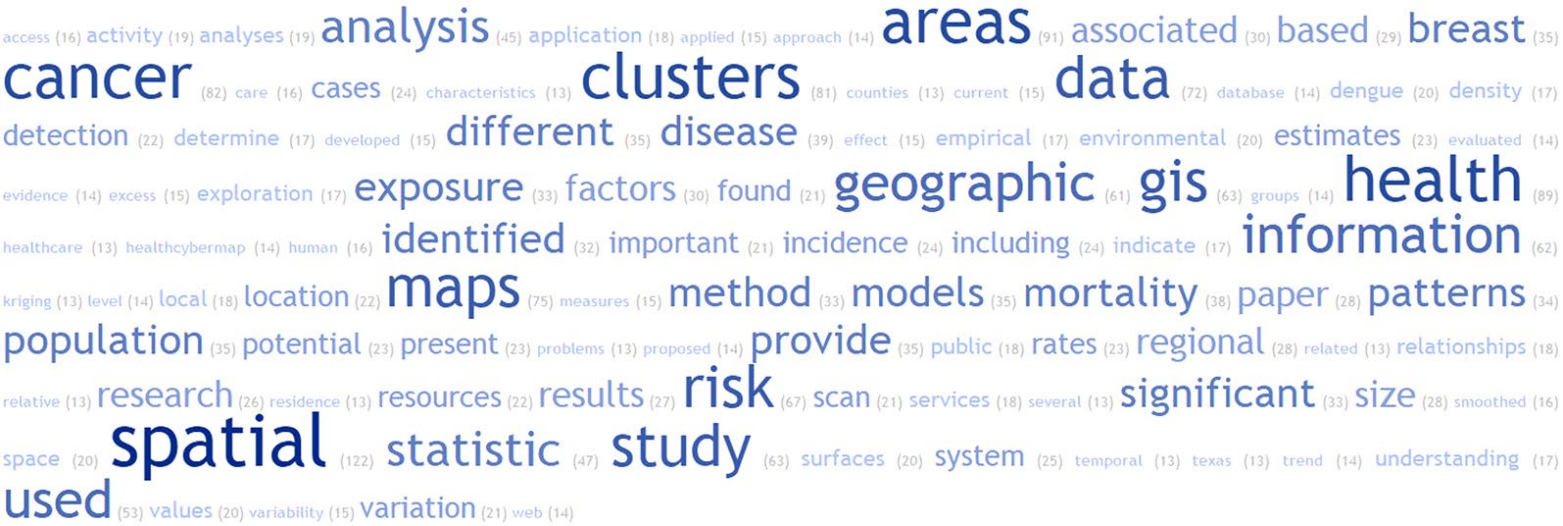
Most used words in the IJHG journal’s articles between 2002 and 2005, the larger the words, the more frequent they are

The tag cloud for the first sub-period (2002–2005) shows that the terms most used in the 43 articles which had been selected are *spatial* (122), *health* (89), *cancer* (82), *clusters* (81) and *maps* (75) (Fig. 1).

Indeed, during the first 3 years of the journal’s existence (2002–2005), the researchers’ attention focused mainly on issues concerning cartography, visualization of data and the use of GIS. Actually, the first article written by the journal’s founder is about retrieving metadata contained in web files in order to produce interactive maps [4], just like another in 2003 by the same author [5]. This goes hand in hand with the georeferencing of data, in particular data on cancer, and the development of spatial analysis tools [6]. In that period, spatial analysis is essentially used with a view to detecting clusters: “We were able to pinpoint geographic areas with higher risk through exploratory spatial analyses, and to assess temporal variability of the risk surfaces, thus providing a working hypothesis on breast cancer and environmental exposures” [7], see also [8]. Then the authors wonder about the best statistical methods for detecting clusters. For example, in the article headed *A flexibly shaped scan statistic for detecting clusters,* Tango and Takahashi question Kulldorff’s statistic which uses a circular window, and put forward another method to approach non-circular clusters (along a road or a river, for example) [9]. Researchers also look into comparing methods and statistical models—*Comparison of spatial scan statistic and spatial filtering in estimating low birth weight* [10], or rather in their combination so as to produce reliable results [11]. GISs are considered as likely to play an important part in following up and understanding the spread of an epidemic of the SARS type in 2003. They allow one to go very far, up to identifying infected buildings that could then be avoided [12].

Spatial clusters are looked for, and so is their temporal inertia. Thus, in *Detecting spatio temporal clusters of accidental poisoning among Texas counties, US, 1980*–*2001,* Nikhoma et al. examine whether clusters vary spatiotemporally when gender and ethnic group are introduced [13]. Results show the persistence through time of the fact that the black population is the most at risk of accidental poisoning, compared to other ethnic groups, and all the more so when the male population is analyzed.

In parallel, warnings against the systematic use of spatial analysis, or at least its limits, appear as early as 2004 [14], with the multi-scale problem (MAUP), the subjectivity of spatial models, looking for associations of variables that do not necessarily imply a causality among them, the fact that there is not such a thing as an absolute model, and that each has its own interests and limits, the ecological inference problem etc.… The very term of “cluster” is challenged, or at least its lack of precision.

### (2006–2010)

**Fig. 2:**
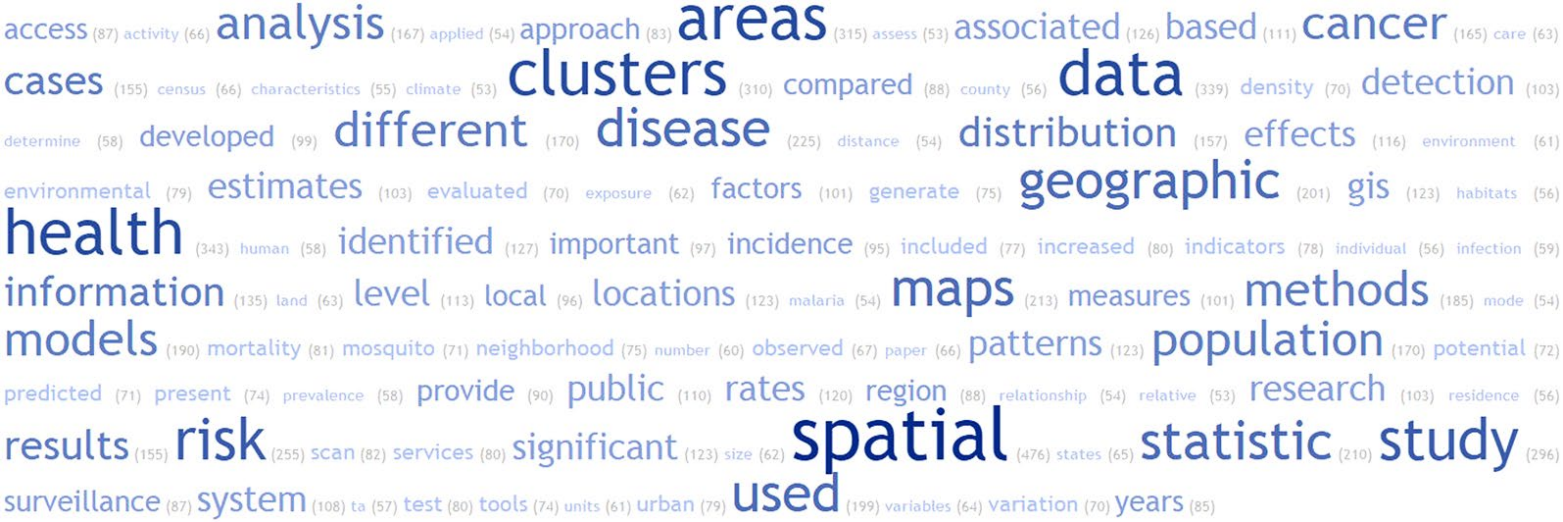
Most used words in the IJHG journal’s articles between 2011 and 2015, the larger the words, the more frequent they are

During the second period (2006–2010), the most frequent words are *spatial* (476), *health* (343) *data* (339), *areas* (315) and *clusters* (310) (Fig. 2). Cluster issues mobilize even more the researchers’ energy (339 against 81), but the link between health geographics and technological progress is increasingly obvious, in particular through geolocation [15, 16]. In parallel, ethics issues and those of right to privacy accompanying these data appear in some articles [17]. Nowadays, the disaggregation of geographic data makes it possible to conduct a reflection on the individual scale, even in the case of GISs [18], since in that article GISs appear in the analysis of the relation between the location of certain lesions and clinical results…

### (2011–2015)

**Fig. 3:**
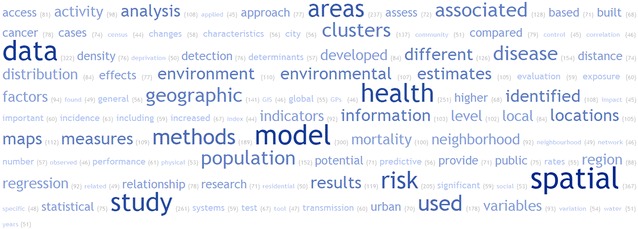
Most used words in the IJHG journal’s articles between 2011 and 2015, the larger the words, the more frequent they are

More recently, between 2011 and September 2015 (147 articles), *spatial* is the most frequent word (367), followed by *data* (322) and *model* (300) (Fig. 3). The question appears of the potentiality of space to influence health, for example through its capacity to favor walking [19], or conversely, environments that are not conducive to that activity and even favorable to falls [20]. Thus, in the article referred to, places conducive to falling down have been identified in a particularly dense Hong Kong neighborhood (MongKok), such as the proximity of underground stations, the junctions of congested streets, traffic lights with too short a time reserved for pedestrians to cross, street edges badly adapted to easy walking, or a sudden and abrupt change of curb. In *Geospatial examination of lithium in drinking water and suicide mortality* [21], it even seems that in some spaces, the suicide rate is lower than national average owing to the natural presence of a certain concentration of lithium in public water, which is a regulator of emotions.

In this lexical study, we have seen the evolution in time of the articles published in the journal. However, although trends can be detected, the articles are highly diversified, which reveals the richness of this field of research. Moreover, space can be approached in very different ways. To make sure of the foregoing, we then selected articles concerning the same research subject, but in which space is not approached in the same way.

The relation to space by different authors can range from space seen as a mere medium for the variables occurring there, to space acknowledged as a proper actor with specific attributes that will influence the phenomenon or the pathology under study either positively (amplification, facilitation…) or on the contrary, negatively (barrier, constraint, attenuation…).

We decided to study the way space is considered via two areas which focus the energy of numerous researchers in Health Geographics, namely vectorial diseases and access to healthcare.

### Vectorial diseases

In the case of articles on vectorial diseases, space is at the same time actor through the numerous determinants (environmental, socio-economic, climatic) that influence the spread of viruses, and medium for the populations or agents (depending on the level considered) implied in the transmission cycles and their interactions. Thus, in *Environmental predictors of West Nile fever risk in Europe* [22], emphasis is put on the role of environmental determinants in the spatial spread of the West Nile virus, a vectorial disease which is transmitted to man via an infected mosquito. Here, environmental determinants designate the physical and climatic characteristics of the spaces being studied. In order to understand the place of these environmental factors, a logistic regression model is used, the dependent variable being the virus infection status (infected/non-infected), and the explanatory variables being environmental determinants provided by teledetection, such as for example the presence of wetlands. The model helps to underline the part of the environment which explains the risk of being infected by the virus. Subsequently, this model can be used as a predictive model so as to assess the probability of future cases. So, here space is perceived as an actor having variable physical and climatic characteristics both in time and space, which plays a determining role in the transmission of the vectorial disease and explains its uneven (spatial and temporal) distribution. Thus, studying the environmental characteristics of the space being studied makes it possible to better understand and predict the spatial variability of the risk of transmission.

In *Agricultural landscape and spatial distribution of Toxoplasma gondii in rural environment: an agent-based model* [23], the authors consider space essentially as a medium of interactions aiming, via multi-agent model, to predict the spatial distribution of a pathogenic factor (difficult to assess in real life), via a number of variables that seem to influence its distribution. Here the parasite is *Toxoplasma gondii*, which is responsible for toxoplasmosis and is excreted in the environment by infected cats (the definitive host) and rodents (intermediate host). The spatial distribution of farms (where cats find shelter) and the distance to the nearest farm could, among others, serve as a medium for the spread of the parasite, and explain high levels of contamination. The risk of contamination is higher if farms are inside a village than if they are scattered. Model entities are cats, rodents, farm buildings and environment cells, each agent and cell being characterized by a state of contamination and specific rules (for example, population dynamic, activity or mobility for the hosts).

In the article by Kienberger et al., *Spatial*-*explicit modeling of social vulnerability to malaria in East Africa* [24], space is perceived both as a medium of the phenomenon being studied and an actor in its representation. This paper is interesting in so far as it proposes an original method to analyze the risk of malaria that can be used as a support for intervention measures. The method is based on a regionalization process. In the paper, results (an index of vulnerability to malaria in the eastern part of sub-Saharan Africa) are visualized by a constructed geographical unit named “geon”. The idea is to offer an alternative to MAUP with a data aggregation method that does not take into account administrative limits or cell grids when data are gridded. The very choice of aggregated data and their regionalization is a process which has a direct influence on the modeling and representation of the variability of the phenomenon being studied.

Unlike vectorial diseases that are not directly transmitted from human to human, interactions such as more or less close contact between healthy and infectious individuals are determining in infectious and contagious diseases [25]. A detailed follow-up of individual behaviors (their mobility in space–time, their exposure to the resulting risk factor) is made possible thanks to geolocation tools. Then the individuals themselves spread the diseases by means of their interactions in their space of activity, and thus create spaces with a more or less high risk of infection.

### Accessibility to health care

The objective is now to synthesize current evidence regarding how space is taken into account in health-related accessibility studies.

Three ecological articles were selected and screened in full text journal. Two articles are from Canada [26, 27], and one article is from France [28]. The aim of the three articles is to assess the spatial accessibility to facilities and more precisely propose the measurement of accessibility on a regional scale using aggregated data in Strasbourg for the methodological article of Salze et al. [28], assess spatial accessibility to healthcare facilities for senior residents in Montreal, in the article of Paez et al. [27], and assess the spatial accessibility to healthy and affordable food in food deserts in a Canadian city for Larsen et al. [26]. For the same objective space was used and analyzed by different means.

Spatial factors have been used to examine how people’s habitual movements interact with their environment. Access is influenced by the shape and area of an individual’s activity space, the spatial distribution of opportunities, and by the spatial structures that constrain and direct movement through space; the shape and area of the activity space is partly a product of how it is conceptualized and measured. Despite being of obvious interest, until recently relatively little was known about the geographical accessibility to healthcare. Whereas Salze and Larsen only take into account spatial factors [28], Paez et al. [27], take into account both individual and spatial factors together.

Two methods were used to assess the spatial accessibility to healthcare: geographic information system (GIS) and spatial analysis with complex models. GIS were used to measure distance between the patient’s location and the facilities. For these measures, they used distance on foot, by car, public transport, and streets. Tools used are mean distance, straight line distance, network based approach [26] or indicators like accessibility or relative accessibility indicators [27]. GISs were used to measure indicators of availability of healthcare facilities using buffers, like the number of facilities taking into account the area around the patient [26]. GISs were used to map the spatial distribution of healthcare facilities and areas of low and high risk of geographical accessibility to healthcare [28]. The second approach is the spatial modeling approach. Salze et al. propose a spatial modeling approach which statistically determines areas of low and high accessibility to a facility in food deserts [28].

### Recent trends

Finally, we wanted to find out what was the latest trend in the last articles. Gong et al.’s article [29] refers to smart space meaning for example “smart city” (see also [30]); there, space is apprehended through a network of economic, smart and communicating sensors able to produce real-time data (coupling GIS and web platform), and warn us when some “event” occurs. Here the term “event” must be understood as a geographic phenomenon occurring at a specific point from a trigger threshold, for example when a certain air pollution threshold is reached. Smart cities were even the subject of a thematic collection of four articles [30–33] published in the journal on January 31, 2015, headed “Smart healthy cities and regions”. What emerges from these is that smart cities, because they are able to act simultaneously on numerous health determinants—for example via a better knowledge of the environment (urban monitoring) and their social action in favor of the elderly (to break their isolation and maintain their autonomy) -, would contribute to reducing health inequalities, even social inequalities among people. Thus, the Internet of Things [31] would be much more than just objects internet-connected through technology, and would make individuals fully in control of their health via, for example, crowdsourcing [32] and feedbacks on noisy environments, love-clean streets, and also more responsible for their health (connected watches, exergames that require physical exercises and make them more attractive for people tending to be sedentary [33]). Cities which are at the same time social, innovative and smart have the capacity to improve their inhabitants’ quality of life and become healthier places where life is pleasant.

Whereas green space, or at least green spaces are seen as contributing too, under certain conditions, not only to our health but also to our overall wellbeing, nevertheless Wheeler et al. [34] underline the importance of carrying out in-depth studies because this relation, which is a priori positive, could not be as linear as one would like to assume…

Finally, the perception of the surrounding space, if it is positive, can both maintain people in good health and avoid to adopt risk behaviors, notably among the youth [35].

This journal specializing in geo-informatics applied to health sometimes deals with non-geographic spaces such as virtual spaces, in order to better figure out, for example, impacts on the health of people living in conflict zones and for whom it is very difficult to have access to data [36], or else space inside our body [18].

## Discussion

Our aim was to focus on the term *space*, and to find out in what way this term and those related were broached in the various articles of the journal in which they appear either in the title or in the abstract. We were particularly interested in the question of what potential has the geographic space to have a direct influence on health matters.

**Fig. 4:**
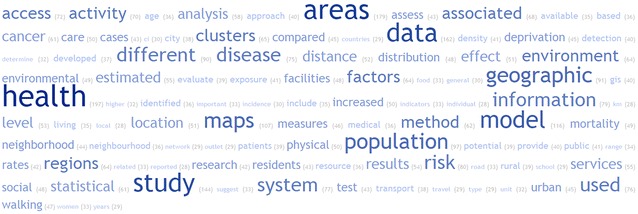
Most used words in the IJHG journal’s articles between 2011 and 2015, the larger the words, the more frequent they are

However, we are well aware that other articles in which these terms appear neither in the title nor in the abstract can nonetheless broach these issues using other words. That is why a lexical analysis has also been done on the articles that had been excluded in order to see whether they were different from those selected. The process was carried out only on articles concerning the most recent period (2011–2015), namely on 108 articles (Fig. 4). What emerges from this analysis is that, most of the time, the words most frequently used are the same as those where the word *space* came out in the forefront, however less frequently, apart from the word *system* which is the most frequently used (77 against 59). But new words appear: *available* (35), *care* (50), c*ountries* (29), *facilities* (48), *food* (33), *individual* (28), *patient* (39), *physical* (50), *school* (29), *services* (55), *social* (48) and *transport* (38).

What are these articles, and in what way do they differ from those previously selected? Some are centered on data-related methodological difficulties, whether these are linked to geo-referencing or cartographic representation, for example they are about how difficult it is to have access to Gis data on a fine scale in some rural regions, and how useful it is then to rely on previously trained community health workers in order to obtain data on the repartition of safe water for people in northern Rwanda [37]. These articles also deal with the development of pertinent cartographic approaches to represent, for example, the risk linked to natural-focal diseases [38], or for better distributing healthcare resources in developing countries [39]. The scale of work seems to be different, the areas being bigger at a regional scale [40] or national [41], it is more about assessing or comparing health systems [42], access to healthcare [43] or health situations [44], transposing methods from a country to another rather than within a limited space where one tries to evaluate one’s ability to act on its inhabitants. The foregoing can partly explain the appearance of the terms *countries*, *facilities* and *services* in these articles.

Other close terms are used in place of the word *space* or the adjective *spatial,* like the word *neighborhood* when the aim is to evaluate the light environment to which people can be exposed, as we know that it may cause circadian disruption due to the level of melatonin [45] or even contribute to the development of breast cancer [46]. The term *neighborhood* is also frequently used to evaluate a food environment [47], or a walkability environment (Neighborhood Environment Walkability Scale-NEWS) [48]. Certain authors resort to this term when studying the impact of a socio-economically disadvantaged environment on the health of its residents [49]. As for the word *area,* it is used in place of the word *space* to observe new geographic areas where certain viruses (arboviruses) spread, triggered by climate changes and notably dryer conditions [50], or to specify the scale of particular studies [51, 52]. Finally, we note the incorporation of *geographical factors* such as altitude, the number of hours of sunshine, relative humidity, temperatures or rainfalls in neural network models in order to measure their impact of the Eyrythrocyte Sedimentation Rate (ESR) [53]. These articles can be backed by cohort studies to find out whether there could be a connection between a long stay in the south-east of the United States and chronic kidney disease, and whether this connection may differ depending on the ethnic group [54].

**Fig. 5:**
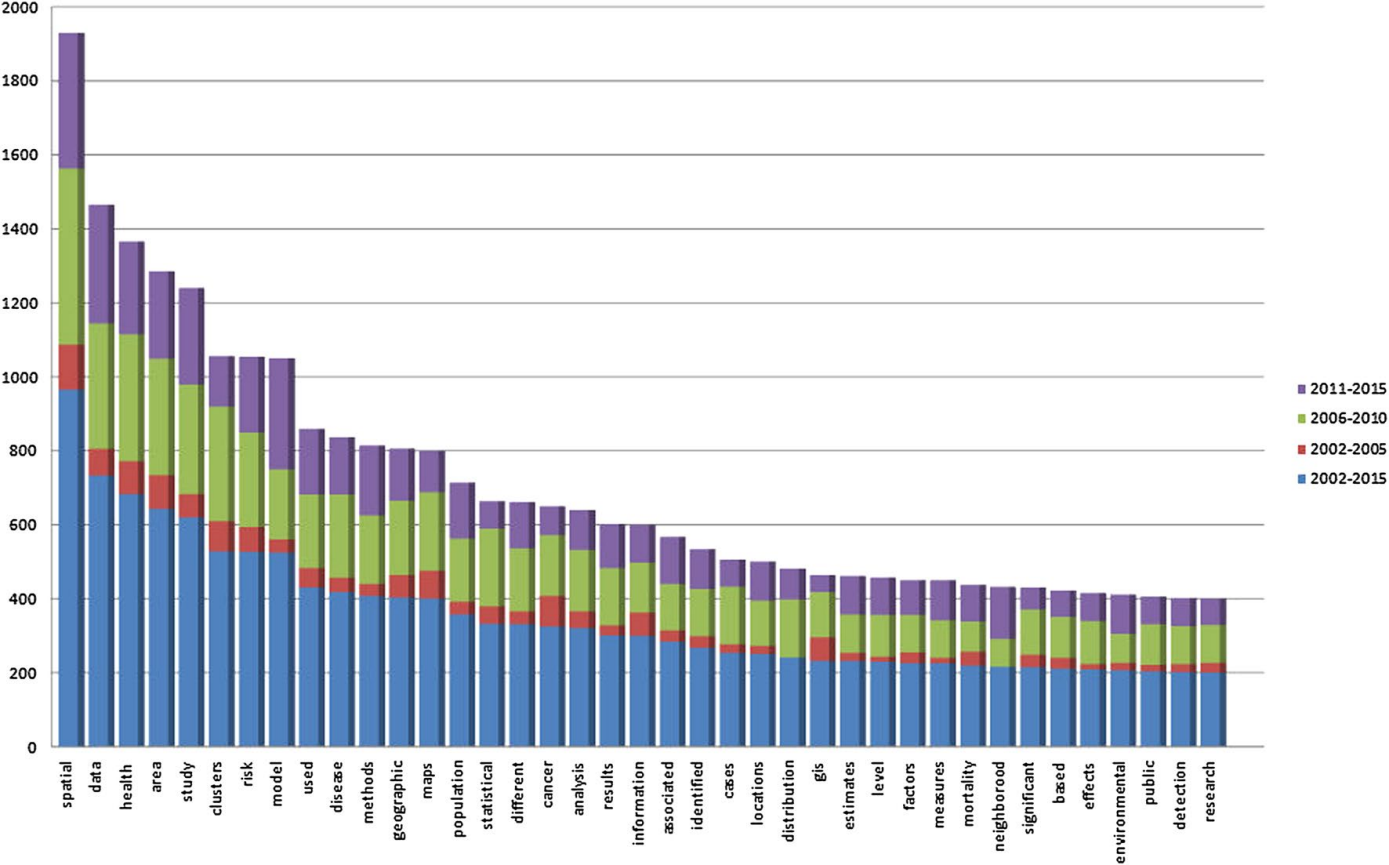
Relative visualisation of the use of the 40 most frequent words in the corpus

Further to these analyses, we wanted to have a relative view of the main words which characterized the different periods, this time in relation to the whole of the articles selected between 2002 and 2015. Because of limitations inherent to TagCrowd, and in particular memory capacity (5 megabyte), it has not been possible to create a tag cloud including all titles and abstracts of all 359 articles selected. Therefore, we have drawn a chart representing the 40 most frequently used words (over 200 times in the whole corpus) which are also found in each period (Fig. 5). This longitudinal view (2002–2015) of the words used in the corpus clearly confirms that the terms most used between 2002 and 2005 (*spatial*, *health*, *cancer*, *clusters* and *maps*) are even most frequently so in the following period (2006–2010), but much less recently (2011–2015). It is interesting to note that only the words *model, neighborhood* and *environment* are used more recently in comparison with other periods. The use of three words fluctuated greatly between 2006 and 2015, the words *cancer* and *clusters* were used less frequently (−87, −173), and conversely the word *model* was used more frequently (+210).

We reach there the limit of this utility; although it has the advantage of giving a general idea of the main words present in a corpus, it nevertheless gives only a snapshot, a frozen image of these words, and the time parameter can only be introduced by splitting the corpus into several periods as we did. Moreover, although it allows to process together words that it deems similar (*space*, *spatial* etc.), it doesn’t allow to select personally from a list of words with a close meaning yet a different root, as we could see with the words *neighborhood*, *area*, *zip codes*, *place*, *location* etc., which are nonetheless related to the geographic space. Furthermore, the words are processed individually and not in context, which may cause a problem in the case of qualifying adverbs and even more with adjacent terms expressing a negation (no, not etc.).

That’s the reason why if we want to go deeper into these analyses, it is preferable to carry out a Keyword in Context analysis (KWIC analysis) using for example a free online tool called Voyant Tools suite (http://voyant-tools.org/). An example of this kind of analysis with free text from the health domain can be found in Maramba et al. [3].

**Fig. 6:**
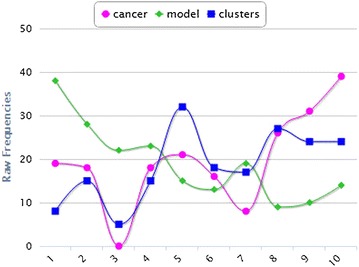
Evolution of the words *cancer*, *model* and *clusters* in the corpus (Voyant tools), warning: the most recent use is the closest to the *Y-axis*: conversely, segment 10 on the *X-axis* corresponds to the oldest articles

**Fig. 7:**
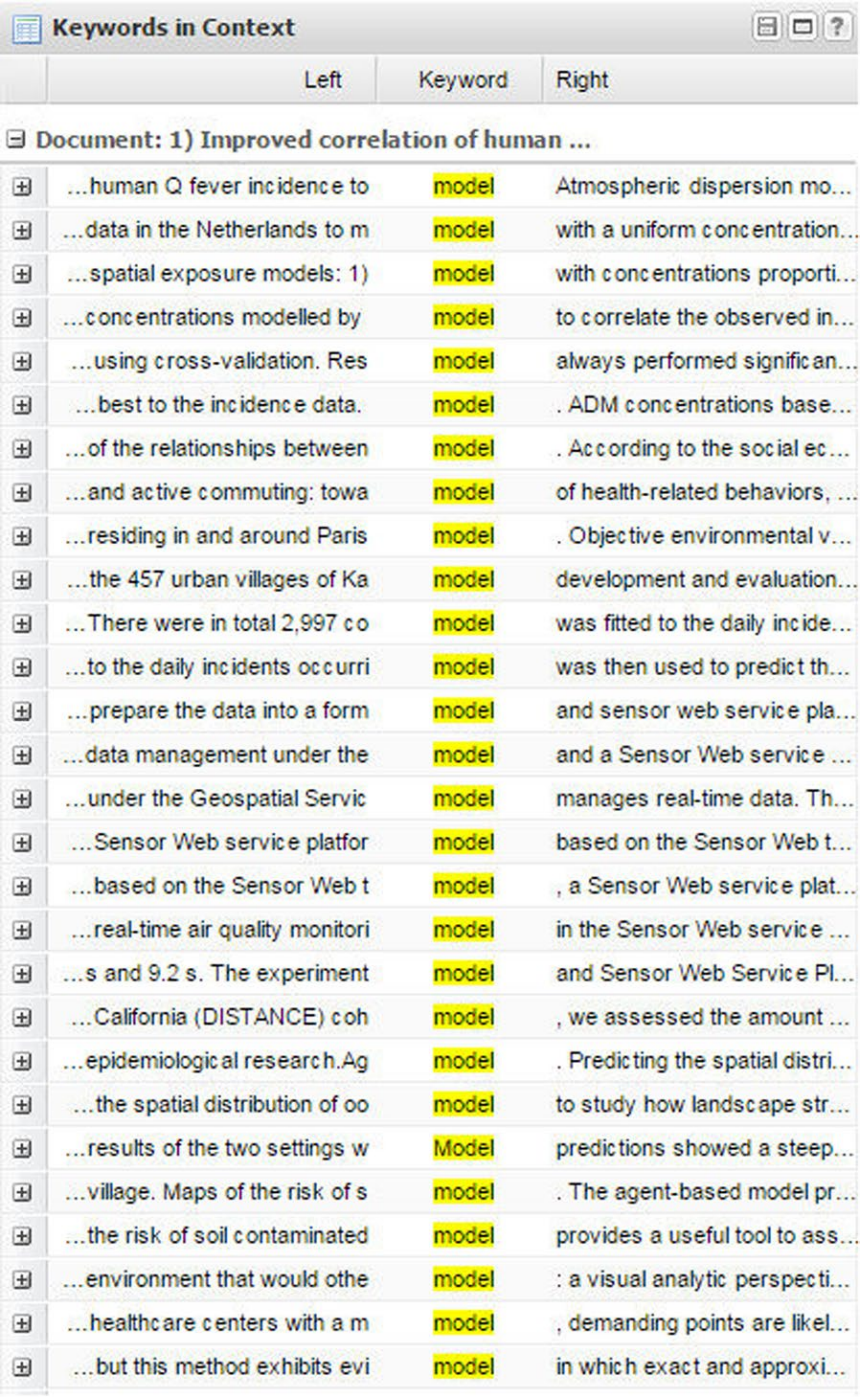
KIWC analysis carried out on the term *model* in the corpus

We used this tool notably to observe more closely the evolution in time of the three terms which evolved most over time (*cancer*, *cluster* and *model*) (Fig. 6). We can see on this chart that although the terms *cluster* and *cancer* have evolved in parallel with the passing time (apart from a slight decoupling between the two curves, where the term *cluster* seems to be used independently from studies on cancer, for example in data relating to congenital malformations [55]), these two terms are less and less used these days, contrary to the term *model* which seems to be used more and more frequently. Indeed, although the detection of clusters is effective for infectious diseases, it seems to be less successful in the case of cancer. This can be explained by the fact that cancer is indubitably a multifactorial disease. Therefore, it is difficult to draw one or several causes that are easily identifiable and explanatory. Thus, researchers may detect cancer clusters in different environmental expositions. Few significant results have come out of such studies [56], and for this reason research on clusters associated to cancer tends to slow down. At present, research extends both to other types of modeling and to numerous themes other than cancer, as shown in Fig. 7 which represents an extract of a KIWC analysis carried out on the word *model,* which contributes to diversifying approaches in health geographics.

## Conclusion

The interest of this contribution was to list a number of general trends that are at the core of health geographics. Health geographics is a recent discipline that relies on new technologies in order to better grasp the links between health facts and their context; it is characterized by a wide methodological diversity and the variety of subtopics broached. Our aim was to emphasize that this field of research is vast, and that beyond the subjects studied, it contributes to a revival of geography via the extension of problems such as MAUP, the visualization of data, issues of ethics and protection of individuals, in particular in regard to the explosion of detailed and geolocated data. In view of the richness of this fast-expanding field, we wished to encourage young geographers to work on these health issues, because there is still so much to do.
